# The prognostic role of soluble TGF‐beta and its dynamics in unresectable pancreatic cancer treated with chemotherapy

**DOI:** 10.1002/cam4.2677

**Published:** 2019-11-07

**Authors:** Hyunkyung Park, Ju‐Hee Bang, Ah‐Rong Nam, Ji Eun Park, Mei Hua Jin, Yung‐Jue Bang, Do‐Youn Oh

**Affiliations:** ^1^ Department of Internal Medicine Seoul National University College of Medicine Seoul Korea; ^2^ Cancer Research Institute Seoul National University College of Medicine Seoul Korea

**Keywords:** biomarker, chemotherapy, pancreatic cancer, prognosis, transforming growth factor‐beta

## Abstract

**Objectives:**

Transforming growth factor‐beta (TGF‐β) is a multifunctional regulatory factor. Here we measured serum soluble TGF‐β (sTGF‐β) levels and evaluated its dynamics and prognostic capabilities during chemotherapy in unresectable pancreatic cancer patients.

**Methods:**

We prospectively enrolled 60 patients treated with FOLFIRINOX as the first‐line palliative chemotherapy. We collected blood samples at the time of diagnosis, first response assessment, and disease progression and measured serum sTGF‐β using an enzyme‐linked immunosorbent assay.

**Results:**

The patients’ median overall survival (OS) and progression‐free survival (PFS) were 10.3 (95% confidence interval [CI], 8.5‐12.1) and 6.5 (95% CI, 4.9‐8.1) months, respectively. Patients with low sTGF‐β at diagnosis (<31.2 ng/mL) had better OS and PFS than patients with high sTGF‐β, respectively, (OS, 13.7 vs 9.2 months; hazard ratio [HR], 2.602; *P = *.004; PFS, 9.0 vs 5.8 months; HR, 2.010; *P = *.034). At the time of disease progression, sTGF‐β was increased compared with that of diagnosis (mean, 26.4 vs 23.9 ng/mL). In particular, sTGF‐β was significantly increased at disease progression in patients with a partial response (mean, 25.7 vs 31.0 ng/mL; *P = *.049).

**Conclusions:**

Pretreatment sTGF‐β levels can serve as a prognostic indicator in unresectable pancreatic cancer patients treated with FOLFIRINOX chemotherapy. Likewise, the dynamics of sTGF‐β during chemotherapy have prognostic value.

## Graphical abstract

Pre‐treatment sTGF‐β levels serve as a prognostic indicator in unresectable pancreatic cancer patients treated with FOLFIRINOX chemotherapy. The dynamics of sTGF‐β during chemotherapy have prognostic value.

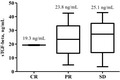

## 1 INTRODUCTION

Pancreatic cancer is a highly lethal malignancy. At the initial diagnosis, only 10‐20% of the patients are candidates for curative resection, as the majority of patients are diagnosed with metastatic or unresectable locally advanced disease.^1^, ^2^, ^3^ In addition, the incidence of recurrence is approximately 80%, even in patients who undergo curative resection; therefore, the 5‐year survival rate is only approximately 10‐20% following the initial diagnosis of resectable pancreatic cancer.^4^, ^5^

Although palliative chemotherapies such as FOLFIRINOX or gemcitabine/nab‐paclitaxel have increased the median overall survival (OS) of metastatic pancreatic cancer patients to 8.5‐11.1 months, the progress of the current treatment modalities is limited by the complex pathogenesis of pancreatic cancer.^6^, ^7^ Many signaling pathways, including the hypoxia‐mediated hedgehog signaling pathway and the stromal cell‐derived factor‐1 (SDF‐1)/C‐X‐C chemokine receptor type 4 (CXCR4) pathway, as well as the accumulation of genetic mutations, contribute to the development of pancreatic cancer.^8^, ^9^, ^10^, ^11^ Inflammation is also relevant to carcinogenesis, and patients who have increased systemic inflammatory markers, such as the neutrophil‐to‐lymphocyte ratio (NLR) and platelet‐to‐lymphocyte ratio (PLR), exhibit a poor prognosis.^12^, ^13^, ^14^

Among the associated mechanisms, the transforming growth factor‐beta (TGF‐β) pathway has been identified as a major contributor to the pathogenesis of pancreatic cancer. The TGF‐β signaling pathway has multifunctional roles in pancreatic cancer, as it acts as both a tumor promoter and a tumor suppressor according to the tumor stage and surrounding microenvironment.^15^ However, in advanced stages, TGF‐β contributes to cancer progression through enhanced invasive properties and the inhibition of immune cell functions.^16^ TGF‐β 1 induces epithelial‐mesenchymal transition (EMT), and it could mediate the transcriptional regulation of EMT‐associated genes through the Smad2/3‐Smad4 complex.^17^ Furthermore, EMT might promote the invasive and metastatic properties of tumor cells.^18^ TGF‐β also contributes to tumor angiogenesis and directly suppresses immune cells.^18^, ^19^, ^20^ Previous studies have reported that increased TGF‐β expression levels in tumor tissues by immunohistochemistry are correlated with poor outcomes in various cancer types.^21^, ^22^, ^23^

A biopsy of the tumor tissue is required to evaluate TGF‐β expression, which is a major limitation to the use of TGF‐β levels in evaluations of the tumor state when needed. Recently, soluble serum biomarkers, such as circulating cell‐free DNA, have been regarded as potential surrogates for predicting clinical outcomes in cancer patients.^24^, ^25^ However, no data on soluble TGF‐β (sTGF‐β) in unresectable pancreatic cancer or its association with prognosis and its dynamic changes during chemotherapy are available.

This study aimed to measure sTGF‐β in the serum of unresectable pancreatic cancer patients treated with palliative chemotherapy and to evaluate the clinical implication of sTGF‐β for predicting survival as well as its dynamics during chemotherapy.

## 2 MATERIALS AND METHODS

### 2.1 Patient characteristics

We prospectively enrolled pathologically diagnosed unresectable pancreatic cancer patients who were treated with FOLFIRINOX as the first‐line palliative chemotherapy at Seoul National University Hospital. Between 2013 and 2015, patients who provided informed consent for the biomarker analysis study were enrolled (n = 60). FOLFIRINOX chemotherapy consisted of oxaliplatin (85 mg/m^2^ for day 1), irinotecan (180 mg/m^2^ for day 1), leucovorin (400 mg/m^2^ for day 1), fluorouracil (5‐FU; 400 mg/m^2^ for day 1), and a continuous infusion of 5‐FU (2400 mg/m^2^ for day 1).

We collected patient clinical information via a medical records review, which included patient demographics, the characteristics of the cancer, and the results of laboratory tests such as total bilirubin, albumin, blood cell counts (neutrophils, lymphocytes, and platelets) and cancer antigen 19‐9 (CA 19‐9) levels. We calculated the NLR and the PLR by dividing the neutrophil or platelet count by the lymphocyte count, respectively. The laboratory test values obtained at the time of the unresectable pancreatic cancer diagnosis were used in the analysis. We evaluated the disease state during FOLFIRINOX chemotherapy using an imaging modality (eg, computerized tomography) every three chemotherapy cycles, and a response assessment was performed according to the Response Evaluation Criteria in Solid Tumors version 1.1.^26^ The responses included complete response (CR), partial response (PR), stable disease (SD), and progressive disease (PD). We defined CR as the disappearance of all target lesions or a reduction in any pathological lymph nodes in the short axis to <10 mm, PR as at least a 30% decrease in the sum of the diameters of the target lesions, and PD as at least a 20% increase in the sum of the diameters of the target lesions compared to the smallest sum during the study. In addition, the appearance of one or more new lesions was also considered progression. We defined SD as neither sufficient shrinkage to qualify for PR nor a sufficient increase to qualify for PD.^26^ We defined the best response as the greatest tumor shrinkage when a response assessment was performed during the FOLFIRINOX chemotherapy.

### 2.2 Measurement of sTGF‐β levels

We prospectively collected blood samples at the time of diagnosis, at the first response assessment (after three cycles of FOLFIRINOX chemotherapy), and at disease progression. Additionally, we evaluated the changes in sTGF‐β levels using paired samples (“at diagnosis and disease progression” or “at first response assessment and disease progression”) and performed subgroup analyses according to the best response during chemotherapy. Paired blood samples at the time of disease progression included samples obtained from patients who developed PD at the time of the first response assessment and who ultimately developed PD after FOLFIRINOX treatment. We measured serum sTGF‐β levels in the patient samples using an enzyme‐linked immunosorbent assay (Human TGF‐beta 1 Quantikine® ELISA Kit, R&D systems) according to manufacturer's instructions.^27^

### 2.3 Statistical analysis

Categorical variables were compared using Pearson's chi‐squared test or Fisher's exact test, as appropriate. Continuous variables were compared using an independent or paired *T*‐test, as appropriate. Progression‐free survival (PFS) and OS were analyzed using the Kaplan‐Meier method. PFS was defined as the time from the initiation of FOLFIRINOX chemotherapy to the date at which disease progression was confirmed by imaging, and OS was defined as the time from the initiation of FOLFIRINOX chemotherapy to the date of either death or last follow‐up. We used a receiver operating characteristics (ROC) curve to determine the cut‐off values of the tumor size and the NLR, PLR, CA 19‐9 levels, and sTGF‐β levels (at diagnosis) to best predict survival. The cut‐off values of other continuous variables were either normal values (albumin and total bilirubin) or median values (age and sTGF‐β levels at disease progression). Clinical variables in the univariate analyses with *P*‐values <0.2 were considered for the multivariate analyses, which were performed using the Cox proportional hazard model. All statistical tests were two‐sided, and significance was defined as *P* < .05. All analyses were performed using IBM SPSS version 22.0 (IBM).

## 3 RESULTS

### 3.1 Patient characteristics

Sixty patients were included in this study. The mean and median values of sTGF‐β were 23.9 ng/mL and 21.7 ng/mL (range, 3.5‐43.0 ng/mL), respectively. We performed a ROC curve analysis to determine the cut‐off values of the tumor size, NLR, PLR, CA 19‐9, and sTGF‐β, and we selected cut‐off values that achieved the highest combination of sensitivity and specificity for the one‐year OS (Table 1).

**Table 1:** ROC curve analysis for the cut‐off point of the variables

Variables	Cut‐off point	Sensitivity (%)	Specificity (%)	AUC	95% CI
Size, cm	5.0	27.4	77.3	0.451	0.333‐0.568
sTGF‐β, ng/mL	31.2	45.7	100.0	0.654	0.518‐0.789
NLR	1.83	89.1	64.3	0.767	0.616‐0.918
PLR	109.6	80.4	50.0	0.626	0.453‐0.798
CA19‐9, U/mL	158.8	69.6	64.3	0.620	0.459‐0.780

Abbreviations: AUC, area under the curve; CA 19‐9, cancer antigen 19‐9; CI, confidence interval; NLR, neutrophil‐to‐lymphocyte ratio; PLR, platelet‐to‐lymphocyte ratio; ROC, receiver operating characteristics; sTGF‐β, soluble transforming growth factor‐beta.

We divided the patients into two groups according to the sTGF‐β cut‐off value; the baseline characteristics of the patients are summarized in Table 2. The patients with higher baseline sTGF‐β levels also exhibited higher CA 19‐9 levels than the patients with lower sTGF‐β levels (81.0% with a high CA 19‐9 ≥ 158.8 U/mL vs 19.0% with a low CA 19‐9 < 158.8 U/mL; *P = *.029). No significant differences according to the sTGF‐β levels were detected for the other clinical characteristics, including age, sex, disease extent, primary site, tumor size, total bilirubin, albumin, NLR, and PLR (all *P > *.05). During the follow‐up period, 2/60 (3.3%) patients achieved CR, 20/60 (33.3%) patients achieved PR, 30/60 (50%) patients exhibited SD, and 8/60 (13.3%) patients exhibited PD as the best response to FOLFIRINOX chemotherapy. The median time from the initiation of chemotherapy to the date of the best response was 5.0 months for CR, 1.7 months for PR, 1.5 months for SD and 1.7 months for PD.

**Table 2:** Baseline characteristics of the patients according to sTGF‐β

Variables		sTGF‐β <31.2 ng/mL (N = 39)	sTGF‐β ≥ 31.2 ng/mL (N = 21)	*P* value
Age, y	≥60	18 (46.2)	7 (33.3)	.337
	<60	21 (53.8)	14 (66.7)	
Sex	Male	25 (64.1)	8 (38.1)	.053
	Female	14 (35.9)	13 (61.9)	
Disease extent	Locally advanced	10 (25.6)	2 (9.5)	.137
	Metastatic	29 (74.4)	19 (90.5)	
Primary site	Head	14 (35.9)	12 (57.1)	.113
	Body or tail	25 (64.1)	9 (42.9)	
Size, cm	≥5.0	11 (28.2)	4 (19.0)	.541
	<5.0	28 (71.8)	17 (81.0)	
CA19‐9, U/mL	Increased (≥158.8)	20 (51.3)	17 (81.0)	.029
	Decreased (<158.8)	19 (48.7)	4 (19.0)	
Total bilirubin, mg/dL	Increased (>1.2)	5 (12.8)	7 (33.3)	.058
	Normal (≤1.2)	34 (87.2)	14 (66.7)	
Albumin, g/dL	Normal (≥3.3)	38 (97.4)	19 (90.5)	.278
	Decreased (<3.3)	1 (2.6)	2 (9.5)	
NLR	Increased (≥1.83)	29 (74.4)	17 (81.0)	.751
	Decreased (<1.83)	10 (25.6)	4 (19.0)	
NLR	Mean	3.00 (±1.55)	2.97 (±1.70)	.940
PLR	Increased (≥109.6)	27 (69.2)	17 (81.0)	.377
	Decreased (<109.6)	12 (30.8)	4 (19.0)	
PLR	Mean	156.6 (±60.01)	171.8 (±72.55)	.388

Abbreviations: CA 19‐9, cancer antigen 19‐9; NLR, neutrophil‐to‐lymphocyte ratio; PLR, platelet‐to‐lymphocyte ratio; sTGF‐β = soluble transforming growth factor‐beta.

### 3.2 Survival outcomes

**Figure 1:**
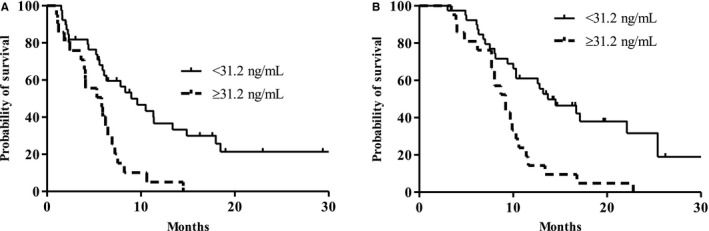
Survival outcomes. A, Progression‐free survival according to soluble transforming growth factor‐beta (sTGF‐β) (median 5.8 vs 9.0 months, *P = *.001). B, Overall survival according to sTGF‐β levels (median 9.2 vs 13.7 months, *P < *.001)

The median follow‐up duration of the 60 patients was 11.4 months (95% confidence interval [CI], 6.9‐14.8 months). The median PFS and OS were 6.5 months (95% CI, 4.9‐8.1 months) and 10.3 (95% CI, 8.5‐12.1 months) months, respectively. The univariate analysis revealed that patients who were older (≥ 60 years) and who had low CA19‐9 levels (<158.8 U/mL), low sTGF‐β levels (<31.2 ng/mL), a low NLR (<1.83), and a low PLR (<109.6) exhibited prolonged PFS (Table 3). In addition, the results showed that age, tumor size, CA 19‐9 level, sTGF‐β level, and NLR were prognostic factors for OS (Table 4). The results of the multivariate analysis revealed that a low sTGF‐β level (<31.2 ng/mL) was an independent prognostic factor for a longer PFS and OS (hazard ratio [HR], 2.010; 95% CI, 1.054‐3.831; *P = *.034 for PFS; HR, 2.602; 95% CI, 1.352‐5.006; *P = *.004 for OS) (Figure 1A,B, Tables 3 and 4). The results also showed that other factors, including increased tumor size (≥5.0 cm), high CA 19‐9 levels (≥158.8 U/mL), and a high NLR (≥1.83), were associated with poor OS (HR, 3.063; 95% CI, 1.493‐6.285; *P = *.002 for tumor size; HR, 2.324; 95% CI, 1.022‐5.285; *P = *.044 for CA 19‐9; HR, 3.451; 95% CI, 1.088‐10.946; *P = *.035 for NLR; Table 4).

**Table 3:** Univariate and multivariate Cox regression analysis for progression‐free survival

Variables		Univariable analysis		Multivariable analysis		
		mPFS (95% CI) (mo)	*P* value	HR	95% CI	*P* value
Age, y	≥60	10.5 (7.0‐14.0)	.026	1		.222
	<60	5.9 (3.7‐8.1)		1.573	0.759‐3.256	
Sex	Male	7.8 (5.0‐10.6)	.464			
	Female	5.9 (4.3‐7.6)				
Disease extent	LAPC	9.0 (3.6‐14.3)	.160	1		.385
	MPC	6.2 (4.5‐7.9)		1.548	0.577‐4.155	
Size, cm	≥5.0	4.4 (2.7‐6.1)	.070	1.866	0.910‐3.828	.089
	<5.0	7.5 (5.0‐10.0)		1		
CA 19‐9, U/mL	≥158.8	5.6 (4.8‐6.4)	.004	1.915	0.875‐4.190	.104
	<158.8	9.0 (4.2‐13.8)		1		
Total bilirubin, mg/dL	>1.2	5.8 (5.1‐6.6)	.448			
	≤1.2	6.9 (5.3‐8.6)				
Albumin, g/dL	≥3.3	Not reached	.428			
	<3.3	6.5 (4.9‐8.0)				
sTGF‐β, ng/mL	≥31.2	5.8 (3.2‐8.5)	.001	2.010	1.054‐3.831	.034
	<31.2	9.0 (5.6‐12.3)		1		
NLR	≥1.83	6.2 (4.8‐7.6)	.008	2.291	0.691‐7.594	.175
	<1.83	Not reached		1		
PLR	≥109.6	6.2 (5.2‐7.3)	.011	1.140	0.410‐3.169	.802
	<109.6	10.5 (0.1‐22.3)		1		

Abbreviations: CA 19‐9, cancer antigen 19‐9; CI, confidence interval; HR, hazard ratio; LAPC, locally advanced pancreatic cancer; MPC, metastatic pancreatic cancer; mPFS, median progression‐free survival; NLR, neutrophil‐to lymphocyte ratio; PLR, platelet‐to‐lymphocyte ratio; sTGF‐β, soluble transforming growth factor‐beta.

**Table 4:** Univariate and multivariate Cox regression analysis for overall survival

Variables		Univariable analysis		Multivariable analysis		
		mOS (95% CI) (mo)	*P* value	HR	95% CI	*P* value
Age, y	≥60	17.1 (0.3‐33.9)	.029	1		.083
	<60	10.3 (8.8‐11.8)		1.933	0.917‐4.075	
Sex	Male	12.6 (8.2‐17.0)	.514			
	Female	10.3 (8.3‐12.3)				
Disease extent	LAPC	16.8 (16.2‐17.4)	.051	1		.911
	MPC	10.0 (9.0‐11.0)		1.055	0.412‐2.701	
Size, cm	≥5.0	6.8 (5.0‐8.6)	.003	3.063	1.493‐6.285	.002
	<5.0	11.6 (8.4‐14.8)		1		
CA 19‐9, U/mL	≥158.8	9.2 (7.2‐11.2)	.003	2.324	1.022‐5.285	.044
	<158.8	17.1 (7.9‐26.3)		1		
Total bilirubin, mg/dL	>1.2	9.7 (6.6‐12.8)	.255			
	≤1.2	11.4 (8.2‐14.6)				
Albumin, g/dL	≥3.3	10.6 (8.7‐12.5)	.802			
	<3.3	9.9 (0.1‐25.7)				
sTGF‐β, ng/mL	≥31.2	9.2 (7.4‐10.8)	<.001	2.602	1.352‐5.006	.004
	<31.2	13.7 (8.3‐19.1)		1		
NLR	≥1.83	10.0 (8.8‐11.2)	.006	3.451	1.088‐10.946	.035
	<1.83	Not reached		1		
PLR	≥109.6	10.3 (8.4‐12.2)	.129	2.282	0.842‐6.182	.105
	<109.6	17.1 (3.7‐30.5)		1		

Abbreviations: CA 19‐9, cancer antigen 19‐9; CI, confidence interval; HR, hazard ratio.; LAPC, locally advanced pancreatic cancer; mOS, median overall survival; MPC, metastatic pancreatic cancer; NLR, neutrophil‐to lymphocyte ratio; PLR, platelet‐to‐lymphocyte ratio; sTGF‐β, soluble transforming growth factor‐beta.

### 3.3 The dynamics of sTGF‐β

All 60 patients provided blood samples at the time of diagnosis, 53 patients provided samples at the first response assessment, and 25 patients provided samples at disease progression. Paired samples, which were obtained “at diagnosis and disease progression” and “at first response assessment and disease progression,” were assessed for 31 patients and 25 patients, respectively.

**Figure 2:**
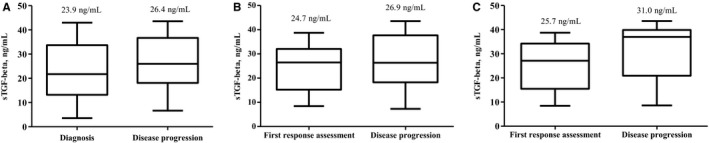
Comparison of soluble transforming growth factor‐beta (sTGF‐β) levels in pancreatic cancer patients during chemotherapy. A, Time of diagnosis vs disease progression (mean 23.9 vs 26.4 ng/mL; *P = *.338). B, First response assessment vs disease progression (mean 24.7 vs 26.9 ng/mL; *P = *.233). C, First response assessment vs disease progression in patients who achieved partial response as the best response during FOLFIRINOX chemotherapy (mean 25.7 vs 31.0 ng/mL; *P = *.049)

The results of a comparison of sTGF‐β levels at the time of diagnosis and at disease progression (n = 31) revealed increased sTGF‐β levels at the time of disease progression versus at the time of diagnosis (mean 26.4 vs 23.9 ng/mL; *P = *.338; Figure 2A). In addition, the results showed increased sTGF‐β levels at disease progression when compared with the levels at the time of the first response assessment (n = 25; mean 24.7 vs 26.9 ng/mL; *P = *.233; Figure 2B). The subgroup analysis results demonstrated that sTGF‐β levels measured at the first response evaluation and again at disease progression were significantly different in patients who achieved PR as the best response during the FOLFIRINOX chemotherapy (n = 10; mean 25.7 vs 31.0 ng/mL; *P = *.049; Figure 2C).

**Figure 3:**
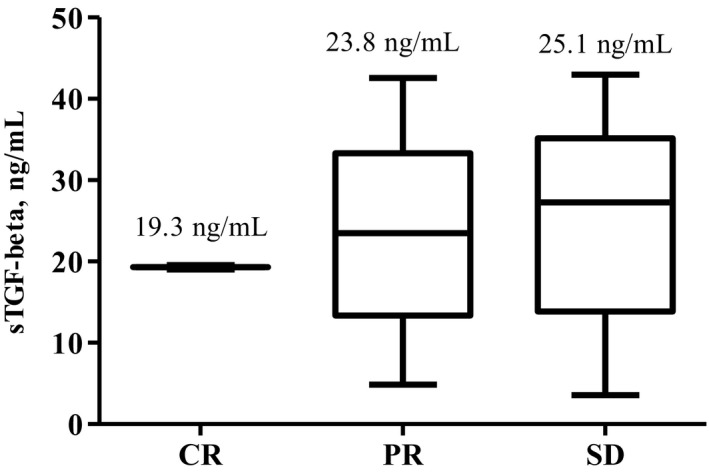
Comparison of soluble transforming growth factor‐beta (sTGF‐β) levels at the time of diagnosis according to the best response during FOLFIRINOX chemotherapy (19.3 ng/mL for CR vs 23.8 ng/mL for PR, *P = *.094; 23.8 ng/mL for PR vs 25.1 ng/mL for SD, *P = *.691; 19.3 for CR vs 25.1 ng/mL for SD, *P = *.011). CR, complete response; PR, partial response; SD, stable disease

When we compared the sTGF‐β levels obtained at diagnosis according to the best response during FOLFIRINOX chemotherapy, we found that patients with poor outcomes as the best response exhibited increased sTGF‐β levels at the time of diagnosis. Patients who achieved CR (n = 2) as the best response showed significantly lower sTGF‐β levels at the time of diagnosis than patients who achieved SD (n = 30) (mean 19.3 vs 25.1 ng/mL; *P = *.011) or PR (mean 19.3 ng/mL for CR vs 23.8 ng/mL for PR; *P = *.094). Additionally, patients who achieved SD as the best response had higher sTGF‐β levels at the time of diagnosis than did patients who achieved PR (mean 23.8 ng/mL for PR vs 25.1 ng/mL for SD; *P = *.691; Figure 3). Survival analyses were performed according to the mean values of sTGF‐β levels (at diagnosis) corresponding to the best response during chemotherapy (19.3 ng/mL for CR, 23.8 ng/mL for PR and 25.1 ng/mL for SD), and the results revealed poor survival outcomes in patients who had increased sTGF‐β levels (median 12.9 vs 0 vs 9.3 vs 9.2 months, *P = *.090, Figure S1A; median 12.9 vs 9.3 months, *P = *.264, Figure S1B; median 12.9 vs 9.3 months, *P = *.022, Figure S1C; median 12.9 vs 9.2 months, *P = *.031, Figure S1D).

The OS from disease progression with FOLFIRINOX to death was also different (high vs low sTGF‐β: median 2.2 vs 4.3 months; *P = *.335) according to the sTGF‐β levels (median 26.4 ng/mL) at disease progression (Figure S2).

## 4 DISCUSSION

The results of the present study indicate that elevated serum sTGF‐β levels at diagnosis predicts poor PFS and OS in unresectable pancreatic cancer patients. In addition, patients with low sTGF‐β levels at diagnosis achieved a better best response during treatment with FOLFIRINOX chemotherapy than did patients with higher sTGF‐β levels at diagnosis.

Previous studies on pancreatic cancer have attempted to evaluate the role of the TGF‐β signaling pathway. One study showed that positive expression of TGF‐β in immunohistochemistry was associated with poor postoperative disease‐free survival in pancreatic cancer patients undergoing surgery (*P* < .05).^23^ Another study revealed that TGF‐β receptor positivity in tumor cells was associated with advanced stage in pancreatic cancer.^28^ However, in many cases, acquiring a sufficient amount of pancreatic tumor tissue to evaluate TGF‐β expression is difficult, which is a major limitation. Recently, serum soluble biomarkers have been regarded as promising indicators and offer several advantages, including minimally invasive collection techniques, cost‐effectiveness, and prediction accuracy.^29^, ^30^, ^31^, ^32^ Therefore, the use of soluble markers could lead to significant advances in our current understanding of cancer dynamics.

The prognostic role of sTGF‐β has been identified in various cancers, including esophageal, bladder, breast, and colorectal cancers.^33^, ^34^, ^35^, ^36^ In locally advanced and metastatic pancreatic cancer, one study reported that patients with higher sTGF‐β levels (≥19.05 ng/mL) exhibited poor OS compared with patients with lower sTGF‐β levels (HR, 1.35; 95% CI, 1.07‐1.69; *P* = .011).^37^ However, in this study, the PFS and the dynamics of sTGF‐β according to disease status were not investigated, which is different from our study.

The present study attempts to illustrate cancer dynamics using the sTGF‐β biomarker. Recently, a few studies have demonstrated the dynamics of soluble biomarkers according to the clinical course in cancer patients. *Ha H* et al revealed that soluble Programmed Death‐Ligand 1 (PD‐L1) was increased in progressive disease compared to baseline or at the time of the best response in biliary tract cancer.^38^ *Thaler FS* et al reported changes in soluble transmembrane activator and CAML interactor (TACI) and soluble B‐cell maturation antigen (BCMA) according to the treatment response in primary central nervous system lymphoma.^39^ In pancreatic cancer, soluble biomarkers have not been well investigated; therefore, our study contributes to the literature by revealing the potential role of sTGF‐β. In our study, although, except for the patients who achieved PR as the best response, there was no significant difference in the sTGF‐β levels between at diagnosis and at disease progression (mean 23.9 vs 26.4 ng/mL, *P = *.338) and between at first response assessment and at disease progression (mean 24.7 vs 26.9 ng/mL, *P = *.233), we demonstrated that sTGF‐β was increased at disease progression compared with that at diagnosis or first response assessment. Additionally, the level of sTGF‐β at the time of disease progression could also predict OS from disease progression after FOLFIRINOX chemotherapy to death, regardless of whether further treatments were administered. Therefore, the results of our study suggest that sTGF‐β can be used as a surrogate for the prediction of disease burden and OS from disease progression to death.

Furthermore, our results showed that an elevated NLR (≥1.83) was associated with a poor OS, and this finding was in accordance with the results of previous studies demonstrating a relationship between increased systemic inflammation and poor outcomes in pancreatic cancer.^12^, ^13^

Our study does have some limitations, as it has small sample size and was performed in a single center. Therefore, significant differences could not be shown in some of the analyses. In particular, we could only confirm that patients who achieved partial response as the best response showed statistically increased sTGF‐β levels at the time of disease progression compared to the first response assessment. To overcome these limitations, additional well‐controlled, large‐scale studies are required to confirm our results. Despite these limitations, to the best of our knowledge, this study is the first to report the dynamics of sTGF‐β in unresectable pancreatic cancer patients treated with homogenous chemotherapy, eg, FOLFIRINOX. In addition, the results of our study demonstrate the potential of sTGF‐β to serve as a powerful prognostic marker for survival outcomes in pancreatic cancer patients, such as the NLR and CA 19‐9 levels.^12^, ^40^ In addition, our study could provide guidance for the selection of patients who could clinically benefit from FOLFIRINOX chemotherapy treatment by measuring sTGF‐β levels at diagnosis.

In conclusion, pretreatment sTGF‐β levels can be used to predict survival outcomes in unresectable pancreatic cancer patients treated with FOLFIRINOX chemotherapy. Likewise, the dynamics of sTGF‐β during chemotherapy have prognostic value.

## CONFLICT OF INTEREST

The authors have declared no conflicts of interest.

## ETHICAL CONSIDERATIONS

This study was reviewed and approved by the Seoul National University Hospital institutional review board (IRB; H‐1307‐146‐507), and was conducted in accordance with the principles established by the Declaration of Helsinki for biomedical research.

## Data Availability

The datasets used during the current study are available from the corresponding author on reasonable request.
