# Supplementary figures and images for: The prognostic role of soluble TGF‐beta and its dynamics in unresectable pancreatic cancer treated with chemotherapy
Source: Cancer Med. 2019 Nov 7;9(1):43–51. doi: 10.1002/cam4.2677 (PMC6943145; doi:10.1002/cam4.2677)

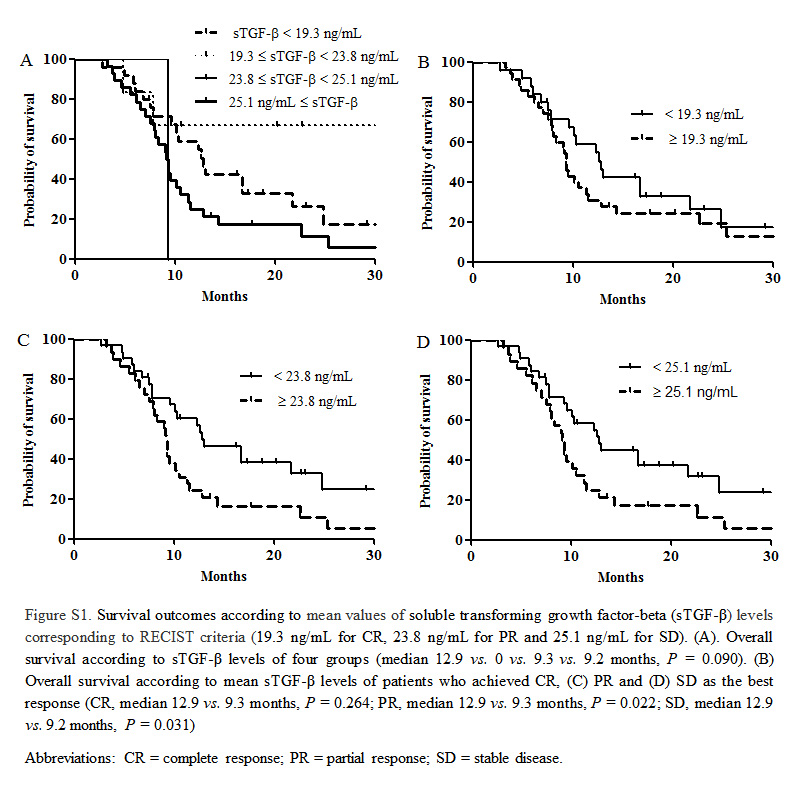

Supplement: Supplementary file 1 [file CAM4-9-43-s001.tif]

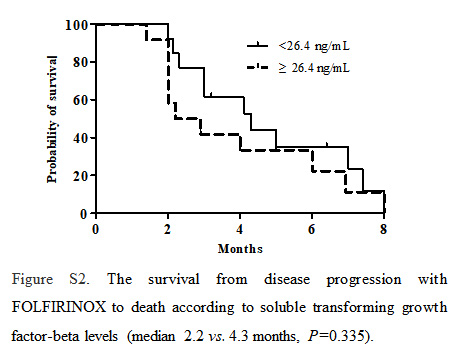

Supplement: Supplementary file 2 [file CAM4-9-43-s002.tif]
